# Leveraging *Pseudomonas* Stress Response Mechanisms for Industrial Applications

**DOI:** 10.3389/fmicb.2021.660134

**Published:** 2021-05-10

**Authors:** Kelly Craig, Brant R. Johnson, Amy Grunden

**Affiliations:** ^1^AgBiome Inc., Research Triangle Park, NC, United States; ^2^Department of Plant and Microbial Biology, North Carolina State University, Raleigh, NC, United States

**Keywords:** stress, *Pseudomonas*, formulation, heat, desiccation, cold, biofilm, chaperone

## Abstract

Members of the genus *Pseudomonas* are metabolically versatile and capable of adapting to a wide variety of environments. Stress physiology of *Pseudomonas* strains has been extensively studied because of their biotechnological potential in agriculture as well as their medical importance with regards to pathogenicity and antibiotic resistance. This versatility and scientific relevance led to a substantial amount of information regarding the stress response of a diverse set of species such as *Pseudomonas chlororaphis*, *P. fluorescens*, *P. putida*, *P. aeruginosa*, and *P. syringae*. In this review, environmental and industrial stressors including desiccation, heat, and cold stress, are cataloged along with their corresponding mechanisms of survival in *Pseudomonas*. Mechanisms of survival are grouped by the type of inducing stress with a focus on adaptations such as synthesis of protective substances, biofilm formation, entering a non-culturable state, enlisting chaperones, transcription and translation regulation, and altering membrane composition. The strategies *Pseudomonas* strains utilize for survival can be leveraged during the development of beneficial strains to increase viability and product efficacy.

## Introduction

Members of the genus *Pseudomonas* have drawn interest for their biotechnology and agricultural potential along with their medical importance as plant and human pathogens. There are 122 recognized and validly published species according to the List of Prokaryotic Names with Standing in Nomenclature (Parte, 2018). Some of the major species groupings are *P. aeruginosa, P. chlororaphis, P. fluorescens, P. putida, and P. syringae* (Gomila et al., 2015). Several species including *P. putida* and *P. fluorescens* have been used for degradation of phenol and other environmental pollutants (Wasi et al., 2013). There are also plant beneficial species such as *P. chlororaphis* that have been utilized as biopesticides to control microbial pathogens, insects, and nematodes (Anderson and Kim, 2018). The plant pathogen *P. syringae* has been studied for its broad host range in commercial crops and the resulting yield reduction (Valencia-Botín and Cisneros-López, 2012). The opportunistic pathogen *P. aeruginosa* is known for infecting immunocompromised individuals and has a natural resistance to many antibiotics (Priebe and Goldberg, 2014).

**FIGURE 1:**
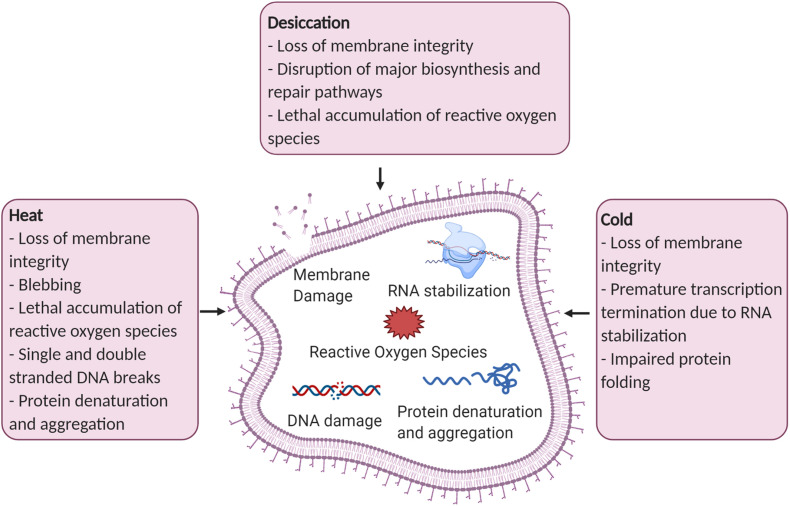
Examples of cellular damage caused by desiccation, heat, and cold stress. Figure created with BioRender.com.

*Pseudomonas* species have been isolated from all over the world from the cold deserts of the trans-Himalayas to deep-sea hydrothermal vents of Juan de Fuca Ridge (Wang et al., 2002; Vyas et al., 2009). *Pseudomonas* species have not only been isolated from extreme environments, but they have also been found to colonize and promote plant growth under extreme temperature and drought conditions (Rolli et al., 2015; Subramanian et al., 2016). *Pseudomonas* strains are exposed to drought, temperature extremes, and many other environmental stressors in nature and have evolved mechanisms to survive these harsh conditions (Figure 1). This review covers the impact of stress exposure on cellular functions and the stress response mechanisms *Pseudomonas* species have adapted to survive these harsh conditions (Table 1). There is a focus on industrially relevant stresses and formulation strategies to improve viability and efficacy during product development.

**TABLE 1:** Overview of bacterial survival mechanisms against desiccation, heat, and cold stress and examples of industrially relevant stress mitigation processes.

Stress	Effect of stress	Mechanism of resistance	Effect of resistance mechanism	Industrially relevant stress mitigation process	References
Desiccation	Damage to cell membrane, accumulation of reactive oxygen species, and loss of protein function.	Accumulation of Compatible Solutes (Trehalose, Alpha-ketoglutarate)	Trehalose replaces water and forms protective matrix. Glutamate eliminates reactive oxygen species.	Addition of protectants for formulation.	Jain and Roy, 2009; Mailloux et al., 2009; Mensink et al., 2017
		Biofilms	Increases persister cells and forms an exracellular matrix barrier.	Fermentation contamination and maintenance.	Lopez et al., 2010
		Exopolysaccharide secretion (Alginate)	Water retention and controls biofilm architecture.	Polymer encapulation for formulation.	Chang et al., 2007; Gulez et al., 2014
Heat	Loss of membrane permeability, DNA damage, and denatured Proteins.	Chaperone (GroEL/GroES or DnaK/DnaJ/GrpE systems)	Correct misfolded proteins.	Regulation of cyclic lipopeptides.	Mayer et al., 2000; Alix, 2006; Georgescauld et al., 2014
		Protease Systems (ClpAP)	Catalyze the breakdown of proteins.	Regulation of cyclic lipopeptides.	Richter et al., 2010; Meyer and Baker, 2011
		Thermosensor (ROSE)	Regulates expression of stress response.	Regulation of rhamnolipid production.	Nocker et al., 2001; Vinella et al., 2005; Narberhaus, 2010
		Alternative sigma factors (sigma 32)	Controls transcription of stress related genes.	Resistance to desiccation in soil environments.	Potvin et al., 2008; Thakur et al., 2013
Cold	An increase in membrane rigidity, over stabilized RNA, and impaired protein folding.	Cold Shock Proteins (Csps and Caps)	Destabilize RNA secondary structure.	Control of post-harvest fungal pathogens during cold storage.	Nakaminami et al., 2006; Trevors et al., 2012
		Antifreeze proteins	Prevents ice crystalization.	Provide freeze protection to freeze sensitive strains.	Venketesh and Dayananda, 2008; Wang et al., 2017
		Membrane Composition (unsaturated fatty acids)	Enhances membrane fluidity.	Provide freeze protection to freeze sensitive strains.	Heipieper et al., 2003; Shivaji and Prakash, 2010
General	General stress response can be triggered by many stressors including desiccation, heat, or cold.	Viable but Non-culturable State	Low metabolic activity for survival.	Inaccurate enumeration of viable cells.	Lowder et al., 2000; Ayrapetyan et al., 2018
		Polyphosphate	Energy storage and stress regulation.	Resistance to nutrient-limiting conditions and elevated temperatures in soil environments.	Nikel et al., 2013; Gray and Jakob, 2014
		Stringent Response	Regulates expression of stress response.	Stress induced tolerance to formulation.	Traxler et al., 2008

## Desiccation Stress

Semi-arid regions cover approximately 15% of the earth’s land surface, and these regions are predicted to continue expanding due to climate change and desertification (Huang et al., 2015). Desiccation stress is frequent in semi-arid regions, and it can temporarily occur in other regions due to seasonal changes, droughts, wet-dry cycles, or a number of other natural causes. Bacteria that are able to survive under these conditions of reduced water availability are called xerotolerant. Some *Pseudomonas* species have moderate resistance to dehydration including many *P. fluorescens* endophytes that colonize roots and have developed mechanisms to persist through drought (Schnider-Keel et al., 2001; Ali et al., 2013; Pravisya et al., 2018).

Bacteria can be exposed to desiccation stress in the natural environment, but this stress can also hinder survival during industrial processes necessary for formulating beneficial, pathogen suppressing bacteria for application onto crops. During the formulation process, products containing beneficial bacteria can be dried to halt metabolism and improve shelf-life stability. Spray drying, fluidized bed drying, and freeze drying are all drying methods to preserve a product through the removal of moisture (Berninger et al., 2018). The stress of dehydration is taken into consideration when selecting a formulation process, and protectants can be added to improve viability (Bashan et al., 2014).

**FIGURE 2:**
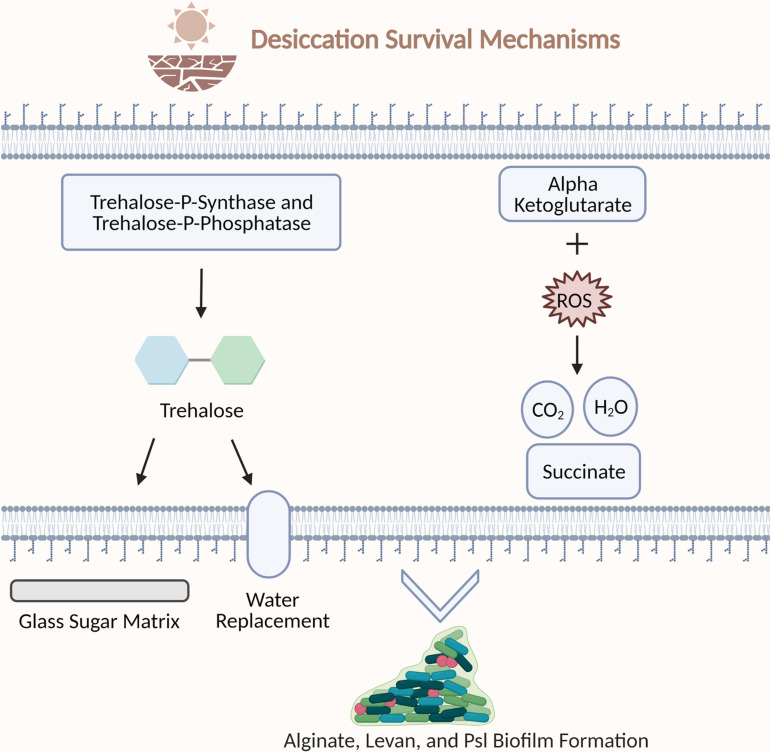
Examples of bacterial strategies to survive desiccation stress. Trehalose-*P*-synthase and trehalose-*P*-phosphatase synthesize trehalose. Trehalose protects the cell by encasing biomolecules in a glass sugar matrix and replacing water hydrogen bonds during desiccation. Alpha ketoglutarate molecules protect the cell by scavenging reactive oxygen species. Alginate, levan, and PSI polysaccharides support biofilm architecture. Figure created with BioRender.com.

Using desiccation as a means to preserve bacteria is a delicate process. Water is critical for survival by providing cell structure and supporting vital molecules including proteins and nucleic acids (Esbelin et al., 2018). Dehydration can result in a loss of membrane integrity, a disruption of major biosynthesis and repair pathways, and a lethal accumulation of reactive oxygen species (Lebre et al., 2017). This common exposure in nature has led to the evolution of desiccation survival mechanisms in bacteria including the production of compatible solutes and biofilm formation (Figure 2).

### Compatible Solutes Overview

Compatible solutes including simple sugars, heterosides, and amino acids are accumulated intracellularly to protect cells from heating, freezing, desiccation, and oxidative stress (Welsh, 2000). Compatible solutes balance osmotic pressure, help maintain cell turgor pressure, can be used as an energy source, and protect cellular structures against changes in water availability. Studies have been conducted on many *Pseudomonas* strains demonstrating their ability to synthesize compatible solutes to overcome different types of stress (Kurz et al., 2010; Sandhya et al., 2010).

### Compatible Solutes – Trehalose

One of the most well studied compatible solutes is the disaccharide trehalose. Trehalose is a stable, colorless non-reducing disaccharide that is used to stabilize products in cosmetics, food, and pharmaceuticals (Schiraldi et al., 2002). There are two proposed mechanisms to explain the desiccation protection properties of trehalose. The first mechanism is that trehalose provides protection by encasing biomolecules in a glass sugar matrix which halts molecular mobility and degradation (Jain and Roy, 2009). The second is the water replacement hypothesis which explains that trehalose protects the native protein conformation during drying by replacing the hydrogen bonds formed between water and the protein with hydrogen bonds formed between the trehalose hydroxyl group and the protein (Mensink et al., 2017).

The ability to accumulate and synthesize trehalose has been identified in *Pseudomonas* and is accomplished through the action of trehalose-P-synthase and trehalose-P-phosphatase enzymes encoded by *otsA* and *otsB* homologs (Lee et al., 2005; Park et al., 2007). The accumulation of trehalose by *Pseudomonas* sp. BCNU 106 was determined by measuring the total intracellular trehalose content, trehalase activity, and mRNA levels of the trehalose-biosynthetic genes (Park et al., 2007). *Pseudomonas* strains have been screened for the presence of trehalose synthase to produce trehalose from maltose. Trehalose synthase has been discovered in *P. stutzeri* and *P. putida* strains, and this enzyme has been cloned and expressed in *Escherichia coli* (*E. coli*) for scaled-up production (Lee et al., 2005; Wang et al., 2014). Trehalose has been demonstrated to protect *Pseudomonas* strains from toxic organic solvents, desiccation, salt stress, and other environmental stressors (Mikkat et al., 2000; Park et al., 2007; Freeman et al., 2010).

### Compatible Solutes – Glutamate

Glutamate and the derivatives of this amino acid play essential roles in nitrogen metabolism and stress tolerance. Alpha ketoglutarate, an intermediate of the TCA Cycle, is utilized by *P. fluorescens* for detoxification of reactive oxygen species (Mailloux et al., 2009). Alpha ketoglutarate eliminates reactive oxygen species by reacting with hydrogen peroxide to form succinate, water, and carbon dioxide (Liu et al., 2018). During oxidative stress alpha ketoglutarate dehydrogenase is downregulated, leaving alpha ketoglutarate molecules to scavenge reactive oxygen species and protect the cell (Mailloux et al., 2009).

Glutamine synthetase was downregulated in *P. syringae* strains in response to osmotic shock (Freeman et al., 2013). Under these conditions, glutamine synthetase would no longer catalyze a conversion of glutamate to glutamine resulting in an accumulation of glutamate. Freeman and associates theorize glutamate may function as a counterion to positively charged potassium ions before transitioning to glutamine synthesis. This hypothesis for glutamates function in osmotic stress has also been demonstrated in studies conducted on the model organism *E. coli* (Cagliero and Jin, 2013). Exogenous potassium ions are brought into the cell to maintain the osmotic or turgor pressure after osmotic stress. To compensate for the net charge increase from the intake of potassium ions, glutamate is accumulated or synthesized so the net charge of the cytoplasm is preserved.

### Formulation Protectants

*Pseudomonas* strains have developed numerous mechanisms to survive exposure to stress in the natural environment. The knowledge gained from studying bacterial mechanisms of survival can be applied to industrial processing. Protectants can be externally applied to beneficial strains to increase viability especially during the formulation process. The formulation process is necessary to enhance the storage capability, transportability, and ease of application in the field. Typically, bacterial strains are grown and harvested from broth, protectants are externally added to the harvested cells, then the cells are dried using methods such as spray drying or freeze drying (Berninger et al., 2018). Protectants can also be added to the medium during growth to be taken up and accumulated in the cell for a protective effect.

There are a number of protectants available including sugars (e.g., sucrose, trehalose, maltose, and lactose), polyols (e.g., mannitol, sorbitol, and glycerol), and amino acids (e.g., L-leucine, Glycine, and L-Proline). Research has been conducted to identify optimal protectants for the formulation of *Pseudomonas* biological control agents. One study was carried out to identify optimal osmoprotectants for three *P. fluorescens* strains capable of reducing Fusarium dry rot of potatoes (Schisler et al., 2016). The osmoprotectants glucose, fructose, trehalose, raffinose, and stachyose were applied to the strains before air drying. Fructose and trehalose were shown to be the most effective osmoprotectants overall though osmoprotectant influence on cell survival varied between strains. Lactose proved to be an optimal lyoprotectant for freeze drying and cold storage of a *P. fluorescens* strain utilized for the control of fire blight disease (Cabrefiga et al., 2014), whereas maltodextrin improved the viability after fluidized bed drying of a *P. protegens* strain capable of controlling bacterial wilt of tomatoes (Wang et al., 2020b). Elucidation of bacterial mechanisms of stress survival has informed the application of protectants during industrial fermentation and formulation processes and is an area of continued research interest for further refining and improving effectual protectant supplementation.

### Biofilms and Exopolysaccharide Secretion

Biofilms are composed of a complex consortium of microorganisms that are adhered to a surface and are encased in an extracellular matrix. The extracellular matrix is composed of extracellular polymeric substances (EPSs) secreted by the cells living in the biofilm such as polysaccharides, proteins, glycoproteins, glycolipids, and extracellular DNA (Flemming et al., 2007; Lopez et al., 2010). The EPS primarily functions to maintain biofilm integrity and to provide protection against desiccation and other stresses. Biofilm formation supports the transition of formerly vegetative cells to non-dividing, antibiotic resistant persister cells, which further enhances their resistance to biotic and abiotic stresses (Lopez et al., 2010).

Polysaccharides are a predominant component of biofilms and help facilitate aggregation, adherence, and surface tolerance (Mann and Wozniak, 2012). Alginate is an exopolysaccharide that plays a major role in *Pseudomonas* biofilm formation by affecting the biofilm structure and stress resistance (Hentzer et al., 2001). Alginate protects cells from water limitation by controlling biofilm architecture and hydration (Gulez et al., 2014). Alginate absorbs and retains water allowing cells to stay hydrated long enough to metabolically adjust to desiccation conditions. Alginate production during water limiting stress in *P. putida* strain mt-2 resulted in taller biofilms covering less surface area with a thicker EPS layer compared to the *algD* mutant strain (Chang et al., 2007). Alginate deficiency was shown to decrease stress tolerance in biocontrol and plant growth promoting strains of *P. putida* and *P. fluorescens* (Svenningsen et al., 2018; Marshall et al., 2019).

Several more polysaccharides are capable of supporting *Pseudomonas* sp. biofilm integrity including levan and the polysaccharide synthesis locus (psl) dependent polysaccharide. Levan has been hypothesized as a source of nutrient stores to protect cells within the biofilm from starvation (Mann and Wozniak, 2012). Studies of *P. syringae* biofilm formation demonstrated an increase in the levan forming enzyme, levansucrase, during planktonic exponential growth and storage of levan in cell-depleted voids within microcolonies (Laue et al., 2006). The *psl*-dependent polysaccharide consists of a repeating pentasaccharide containing D-mannose, D-glucose, and L-rhamnose (Byrd et al., 2009). The deletion of *psl* genes has been demonstrated to dramatically decrease biofilm formation in *P. aeruginosa* (Ma et al., 2012). In addition, the comparison of alginate or Psl producing strains revealed that overproduction of alginate leads to mucoid biofilms, which occupy more space, while Psl-dependent biofilms are densely packed.

### Biofilm Disruption of Formulation Methods

Detrimental biofilms have become a major problem in healthcare, food, and agricultural industries. The resilience of biofilms to stress has led to significant issues with contaminants persisting through sterilization procedures. *P. aeruginosa* has caused persistent infections in immunocompromised patients through biofilm formation on medical equipment (Hadi et al., 2009). *P. syringae* has been isolated from irrigation water and epilithic biofilms (Morris et al., 2008). Biofilms can become the source of infection in agricultural fields by contaminating irrigation systems with plant pathogens (Raudales et al., 2014). In addition, biofilms can build up in pipes, tubing, and tanks causing persistent contamination during the fermentation and processing of microbial products (Rich et al., 2015). Many strategies including chelating agents, peptide antibiotics, lantibiotics and synthetic chemical compounds have been developed to control biofilms in industrial processing (Roy et al., 2018).

### Alginate Encapsulation

Biological control agents can be encapsulated by surrounding the bacteria with coats of material such as polymers to create small capsules (Huq et al., 2013). Encapsulation can be used to protect biological control agents from stress and slowly release the bacteria over longer periods of time. Alginate is a common material for encapsulation because it is biodegradable, non-toxic, and easy to handle (Power et al., 2011). Several *P. fluorescens* strains have been encapsulated with alginate formulations to study the impact on storage survival and pathogen control (Pour et al., 2019; Kadmiri et al., 2020). *P. fluorescens* strains VUPF5 and T17-4 were encapsulated with an alginate-gelatin formulation which improved shelf life and control of dry rot induced by *Fusarium solani* under greenhouse conditions (Pour et al., 2019). The microbial fertilizer *P. fluorescens* Ms-01 was encapsulated with halloysite and alginate or montmorillonite and alginate formulations (Kadmiri et al., 2020). Both formulations were able to preserve bacterial survival after 3 months storage at room temperature or 4°C. Encapsulation of microorganisms in a semipermeable membrane separates and protects the cells from the surrounding environment (Pour et al., 2019). This method protects biological control agents from abiotic stresses allowing proliferation in the environment and effective deployment against plant pathogens.

## Heat Stress

Bacteria are exposed to heat stress in the natural environment through various events including changes in weather and host responses. *Pseudomonas* species are typically mesophiles growing from a range of 20–45°C, but there have been a few instances of thermotolerant species such as *P. thermotolerans* (Manaia and Moore, 2002). The opportunistic pathogen *P. aeruginosa* can infect immunocompromised individuals and has been isolated from patients experiencing fevers over 38°C (Han et al., 2019). *Pseudomonas* strains have been discovered all over the world including areas on the equator with elevated temperatures (Fernandes et al., 2018; Eheth et al., 2019).

With advancements in food safety and other fields of research, bacteria are exposed to extreme temperatures during the manufacturing of products. Heat treatments involved in pasteurization and sterilization have widely been used for food safety (Cebrián et al., 2017). Several *Pseudomonas* species including *P. lundensis* and *P. fragi* have heat-resistant proteases responsible for the spoilage of dairy products (Marchand et al., 2009; Stoeckel et al., 2016). Similarly, beneficial bacteria are exposed to heat during the formulation process to create an easily distributed and shelf-stable dried product. During this process bacteria could be spray dried at temperatures above 100°C, but there are less harsh options available such as freeze drying that may be more suitable for temperature sensitive strains (Anekella and Orsat, 2013).

**FIGURE 3:**
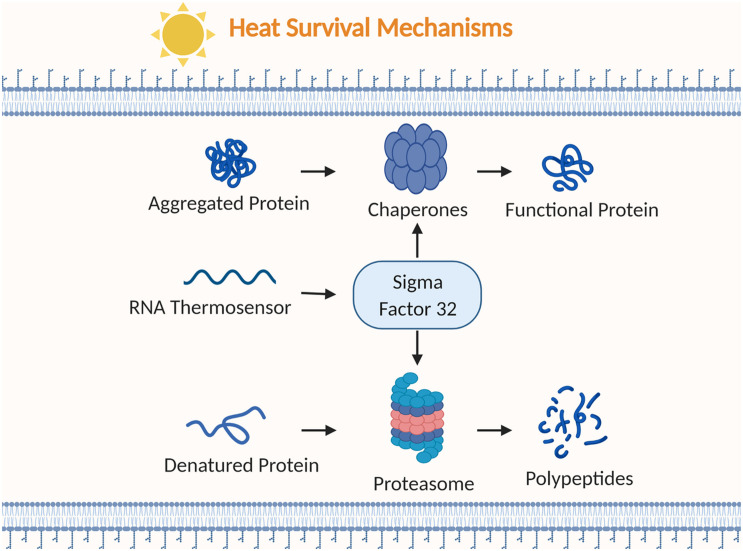
Examples of bacterial strategies to survive heat stress. Chaperones alter aggregated or misfolded proteins into functional proteins. RNA thermosensors regulate the heat shock sigma factor (σ32). Proteasomes break down denatured proteins into polypeptides. Figure created with BioRender.com.

Heat stress affects many critical bacterial structures including the membrane, DNA, and proteins. Damage to lipopolysaccharides compromises outer and cytoplasmic membrane structures resulting in blebbing, meaning protrusions of the outer membrane, and a loss of permeability (Russell, 2003). The loss of permeability can lead to a leakage of intracellular compounds and an inability to transport substances into the cell. DNA can be impacted from heat exposure by an increase in mutation frequency and both single and double stranded DNA breaks (Cebrián et al., 2017). Protein denaturation is another potentially lethal effect of heat stress leaving structural proteins and enzymes damaged. *Pseudomonas* strains have evolved to rapidly respond to the harsh effects of heat stress through production of chaperones and proteases, and regulating with thermosensors and alternative sigma factors (Figure 3).

### Chaperone Overview

Bacteria must make rapid responses to the changing environment by forming and replacing cellular proteins. Molecular chaperones are proteins that play a critical role in this process by assisting with the correct folding and assembly or disassembly of other proteins (Lawless and Hubbard, 2014). Chaperones remain important under non-stressed conditions as self-assembly of some proteins occur at slower rates and can lead to misfolding or aggregation of partially folded intermediates (Alix, 2006). A rapid increase in chaperone synthesis occurs under heat and other stress conditions to control the aggregation of denatured proteins.

### Chaperone – GroEL/GroES

GroEL and DnaK are two well characterized chaperone systems conserved throughout many bacteria (Singh and Gupta, 2009). The heat shock protein system GroEL/GroES consists of the 60 kDa chaperonin GroEL that forms two stacked rings in a barrel-like structure and a 10 kDa cofactor GroES that forms a single ring structure (Alix, 2006). An unfolded protein binds to the hydrophobic amino acid residues on the interior rim of the GroEL ring opening (Georgescauld et al., 2014). ATP binds to GroEL inducing a conformational change allowing GroES to attach and close off GroEL. This formation results in an enclosed cage with a hydrophilic interior leading the unfolded protein to release from the rim and refold. ATP hydrolysis and protein binding to the opposite ring causes GroES and the protein to release.

Research on heat shock response in *Pseudomonas* has revealed a GroEL/GroES system similar to the system found in the model organism *E. coli* (Ito et al., 2014). The GroES/GroEL system was found in *P. aeruginosa* PAO1 through S1 nuclease mapping and northern hybridization with a two-fold upregulation under heat shock (Fujita et al., 1998). The *groES* and *groEL* region is conserved amongst *Pseudomonas* species. In fact, the 536 bp region of the *groE* gene was used to develop a diagnostic PCR assay for detection of *P. aeruginosa*, *P. putida*, *P. fluorescens*, and *P. stutzeri* (Clarke et al., 2003).

### Chaperone – DnaK/DnaJ/GrpE System

The DnaK, DnaJ, and GrpE chaperone system prevents proteins that are damaged or stuck in an intermediate folded state from aggregating. The system consists of the chaperone DnaK, the co-chaperone DnaJ, and the nucleotide exchange factor GrpE. DnaK is a 70 kDa chaperone with an ATP-binding N-terminal, a substrate binding domain, and a peptide binding C-terminal domain (Mayer et al., 2000). The hydrophobic amino acid residue of misfolded proteins will bind to the substrate binding domain of DnaK. DnaJ will promote hydrolysis of ATP to ADP at the N-terminal ATPase domain (Alderson et al., 2016). When ADP is bound, the C-terminal domain locks down on the protein by closing off the substrate-binding site. DnaJ dissociates and the complex will continue to hold the protein. GrpE binds leading to a conformational change that releases ADP and shifts DnaK to an open conformation.

Several studies have been conducted to better understand the role of DnaK in the *Pseudomonas* heat shock response. Transcriptome analysis was performed on *P. aeruginosa* PAO1 grown at human body temperature (37°C) and an elevated temperature (46°C) (Chan et al., 2016). RNA sequencing revealed 133 genes were differentially expressed including the upregulation of *dnaK*, *dnaJ*, and *groEL* at 46°C compared to human body temperature. The heat shock response of *P. putida* KT2442 was elucidated by constructing and testing null mutants of molecular chaperone genes (Ito et al., 2014). The *P. putida* KT2442 *dnaJ* mutant was temperature-sensitive and formed more protein aggregates in response to heat stress compared to the wild type.

### Protease Overview

Proteases are crucial for rapid responses to environmental changes when damaged proteins cannot be salvaged by chaperones or other heat shock proteins. Proteases are enzymes that catalyze the breakdown of proteins into amino acids or smaller polypeptides by cleaving peptide bonds. They contribute to the heat shock response by removing misfolded or damaged proteins and recovering the amino acids (Meyer and Baker, 2011). Proteases will also degrade functional proteins such as sigma factors, metabolic enzymes and structural proteins that are no longer needed as cells adapt to changes (Kirstein et al., 2009; Bittner et al., 2016). The Clp protease family is a highly conserved system that is vital for stress survival in many bacteria, including *Pseudomonas* species.

Clp complexes contain chaperones and proteases that belong to the heat shock protein family HSP100 and the ATPases Associated with diverse cellular Activities (AAA+) protein superfamily. The AAA+ superfamily has a highly conserved AAA+ module consisting of 250 amino acids, and they typically have motifs involved in ATP binding and hydrolysis (Ogura and Wilkinson, 2001). ATP-dependent Clp protease (ClpP) consists of a ClpP serine peptidase subunit and a AAA+ ATPase subunit such as ClpA, ClpC, or ClpX (Richter et al., 2010). ClpP forms a barrel-like structure with two stacked heptameric rings of the ClpP serine peptidase subunit and is enclosed by one or two hexameric rings of the AAA + ATPase subunit. The Clp ATPase subunit uses ATP hydrolysis to unfold protein substrates then directs them through a central pore to the proteolytic chamber of ClpP for degradation (Snider et al., 2008).

Clp Peptidase has been found to control diverse aspects of cellular physiology in *Pseudomonas* species. The proteolysis activity of ClpP helps regulate the stress response sigma factor RpoS (σ38) and other transcriptional regulators influencing many factors including motility, surface attachment, and antimicrobial activity (Bruijn and Raaijmakers, 2009). *P. aeruginosa* was found to have a second ClpP isomer which impacts stationary-phase cells, microcolony organization and biofilm formation (Hall et al., 2017). *ClpP, clpX*, and *clpP2* null mutants of *P. aeruginosa* PAO581 were created to study the regulation of exopolysaccharide alginate overproduction and the conversion to a mucoid phenotype which are markers for the onset of chronic lung infection in cystic fibrosis patients (Qiu et al., 2008). All null mutants demonstrated a decrease in transcriptional activity of the extracytoplasmic function sigma factor AlgU which is responsible for alginate overproduction. The *clpP* and *clpX* null mutants were unable to maintain the mucoid phenotype.

### Regulation by Chaperones and Proteases

Chaperones and Proteases such as DnaK and Clp control the regulation of cyclic lipopeptides in many *Pseudomonas* species. Cyclic lipopeptides have a role in biofilm formation, surface motility, and antimicrobial activity (Dubern et al., 2005). Putisolvin and massetolide have activity against oomycete plant pathogens by causing lysis of zoospores (Raaijmakers et al., 2010). The DnaK complex is hypothesized to regulate putisolvin biosynthesis by controlling activity of other regulators or assisting with the assembly of putisolvin peptide synthetases. ClpP regulates massetolide biosynthesis by degradation of putative transcriptional repressors of massetolide biosynthesis genes (Bruijn and Raaijmakers, 2009). A range of growth temperatures (32, 28, 21, 16, and 11°C) were tested on a *P. Putida* strain and three *dnaK*, *dnaJ*, and *grpE* mutants to determine the effect on putisolvin production (Dubern et al., 2005). It was shown that putisolvin production decreased at high growth temperatures, but putisolvin production increased at low temperatures. DnaK and DnaJ are required to produce putisolvins at low temperatures. These studies indicate that growth temperature can influence DnaK and DnaJ regulation of putisolvin synthesis, and growth at low temperatures may enhance putisolvin production. Chaperones and proteases are key components to survival and efficacy for many *Pseudomonas* species. Understanding regulatory effects of these proteins is crucial to the development of *Pseudomonas* strains as biocontrol products.

### Thermosensors

Adverse environmental conditions such as elevated temperatures can damage proteins requiring molecular chaperones or proteases to help repair cells. Thermosensors are RNA, DNA, or protein molecules that react to changes in environmental conditions and regulate gene expression of the heat shock response and other stress responses. Temperature shifts cause thermosensors to change their secondary structure, and this change typically either exposes or blocks nucleic acid regions influencing expression of a gene. DNA topology can act as a stress sensor because transcription efficiency is sensitive to changes in DNA supercoiling (Klinkert and Narberhaus, 2009). RNA thermosensors are translational control elements that are mostly located in the 5′ untranslated region (UTR) of messenger RNA encoding heat shock proteins (Narberhaus, 2010). There are a diverse set of protein thermosensors including transcriptional repressors, sensor kinases, chaperones, and proteases (Collin and Schuch(eds), 2009). The diversity is due to the sensitivity of tertiary and quaternary protein structures to temperature stress (Klinkert and Narberhaus, 2009).

Repression of heat shock gene expression (ROSE) elements are RNA thermosensors located in the 5’ UTR of some heat shock protein mRNA (Nocker et al., 2001). ROSE elements structures consist of two to four stem loops and they are characterized by a conserved G residue that pairs with the Shine Dalgarno sequence (AGGA) (Klinkert and Narberhaus, 2009). Under normal conditions, the conserved G residue opposite the Shine Dalgarno sequence closes the loop by a weak GG base pair preventing gene expression. The weak GG base pair breaks under temperature stress allowing the ribosome binding site to be accessed for translation.

Structural and functional analysis has uncovered the ROSE elements’ role in heat shock and virulence regulation for *Pseudomonas* species. *P. putida* and *P. aeruginosa* contain a simpler ROSE element with only two hairpins compared to previously discovered *Rhizobium* sp. ROSE structures with three to four hairpin structures (Nocker et al., 2001; Krajewski et al., 2013). The ROSE element precedes an *ibpA* gene which encodes the small heat shock protein IbpA (inclusion body associated protein A). High sequence conservation of *ibpA* untranslated regions suggests ROSE thermosensors could be common for IbpA regulation in *Pseudomonas* species. ROSE elements have also been studied for the temperature induced regulation of rhamnolipid production in *Pseudomonas* species. Bioremediation strains of *Pseudomonas* produce rhamnolipids which facilitate the uptake and degradation of hydrocarbons such as crude oil in polluted environments (Chrzanowski et al., 2012). *P. aeruginosa* is one of the most competent rhamnolipid producers and safe methods to produce rhamnolipids are being explored including strain engineering to diminish pathogenicity (Chong and Li, 2017). *P. putida* KT2440 rhamnolipid production increased by more than 60% when growth temperature was increased from 30 to 37°C (Noll et al., 2019). These studies demonstrate the potential to design a process for rhamnolipid production linked to temperature and ROSE regulation.

### RNA Polymerase Sigma Factors

Sigma factors are essential for regulation of both essential genes and conditional responses such as heat or cold shock. A sigma factor is a protein required for transcription initiation that allows RNA polymerase to bind to specific gene promoters. Primary sigma factor (σ70) is named for its 70 kDa molecular weight, and it is responsible for regulation of essential genes during exponential growth. Sigma factor 70 binds to RNA polymerase allowing promoter recognition at —10 and —35 nucleotides upstream of the start site (Potvin et al., 2008). Alternative sigma factors are needed for transcription of genes linked to environmental or physiological changes. In response to stress RNA polymerases are redirected by alternative sigma factors to activate transcription of genes to help the cell respond to new conditions.

Genome analysis of *P. aeruginosa* PAO1 and *P. syringae* pv. syringae B728a revealed the presence of 24 and 15 putative sigma factors, respectively, including primary sigma factor (σ70), heat shock sigma factor (σ32), stationary phase sigma factor (σ38), and nitrogen-limitation sigma factor (σ54) (Potvin et al., 2008; Thakur et al., 2013). The majority were classified as extracytoplasmic function (ECF) sigma factors which are involved in the regulation of a diverse set of functions including iron starvation, cell envelope stress, solvent tolerance, and oxidative stress (Otero-Asman et al., 2019). Stationary phase sigma factor RpoS (σ38) is a master stress response regulator active during stationary phase to protect against heat shock, starvation, and osmotic shock (Schuster et al., 2004). The heat shock sigma factor RpoH (σ32) was upregulated as temperature increased from 30 to 42°C in *P. aeruginosa* (Nakahigashi et al., 1998). RpoH regulates heat shock genes responsible for the expression of chaperones and proteases such as GroEL, GroES, DnaK, DnaJ, GrpE, and ClpP (Aramaki et al., 2001; Potvin et al., 2008). Sigma Factor AlgU (σ22) mutant strains of *Pseudomonas fluorescens* CHA0 were studied to determine the impact of AlgU on environmental stress survival (Schnider-Keel et al., 2001). Schnider-Keel et al. (2001) found that AlgU is critical for survival against desiccation and hyperosmolarity in soil environments. Alternative sigma factors are critical to survival against major industrial and environmental stresses.

## Cold Stress

Cold climates are widespread on earth, with ice covering 10% of the earth’s surface and a large percentage of the biosphere existing at temperatures below 15°C (Kuhn, 2012). Bacteria that can colonize these extreme environments and grow at –20°C to 20°C are called psychrophiles (De Maayer et al., 2014). Several *Pseudomonas* psychrophiles have been isolated from glaciers in Antarctica and the Arctic Circle such as *P. antarctica* (Villeret et al., 1997; Dziewit et al., 2013; Lee et al., 2017). Cold stress survival is of particular importance for the control of post-harvest fruit and vegetable diseases. Many *Pseudomonas* species are adapted to cold stress and effectively control post-harvest fungal pathogens during cold storage (Wallace et al., 2017; Aiello et al., 2019). The strain *P. syringae* ESC-10 was able to survive 30 days of 2°C cold storage and prevented post-harvest blue mold on apples (Errampalli and Brubacher, 2006). Many strains of *Pseudomonas*, such as *P. syringae* ESC-10, are psychrotrophs or mesophiles that grow at more moderate temperatures and have adapted mechanisms to survive exposure to cold stress.

Freezing cultures, using processes such as freeze drying, is one of the most common methods for preserving bacterial inoculants. Freeze drying is a freezing and dehydration preservation technique that involves freezing, lowering pressure and removing ice through sublimation (Fellows, 2009). The food industry and the pharmaceutical industry rely on freeze drying for starter cultures, drug delivery, and long-term storage in culture collections. Manufacturing of commercial foods requires vast amounts of starter cultures with cheese production alone requiring approximately one billion liters of bulk starter per year (Champagne et al., 1991). Freeze drying of biocontrol *Pseudomonas* strains has been explored as a method to deliver viable cells for crop protection (Cabrefiga et al., 2014).

**FIGURE 4:**
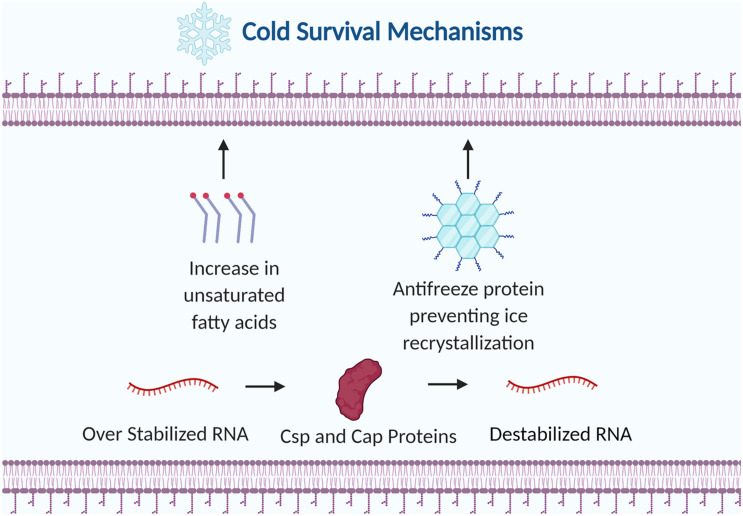
Examples of bacterial strategies to survive cold stress. The membrane is altered by increasing the ratio of unsaturated fatty acids to saturated fatty acids. Antifreeze proteins are produced to prevent ice recrystallization. Cold shock proteins (Csps) and cold adaptive proteins (Caps) destabilize RNA to prevent premature transcription termination. Figure created with BioRender.com.

Cold stress drastically affects cellular physiology by damaging the cell membrane, impairing protein folding and hindering transcription and translation (Kumar et al., 2020). Rapid chilling can induce phase separation of phospholipids within the lipid bilayer leading to membrane permeability and consequently cell death (Cao-Hoang et al., 2008). At low temperatures the secondary structures of RNA stabilizes which slows down transcription elongation and ribosomal movement on RNA (Phadtare and Inouye, 2004). Cold tolerant microbes can survive these harsh conditions by adjusting the fatty acid composition in the membrane, producing specialized enzymes that are active at low temperatures, and by creating cryoprotective biomolecules (Figure 4).

### Cold Shock Proteins and Cold Acclimation Proteins

Cold shock proteins (csp) and cold acclimation proteins (cap) are two strategies *Pseudomonas* strains utilize to adapt to cold environments. Genes encoding cold shock proteins are expressed immediately after cold shock while genes encoding cold acclimation proteins are expressed during prolonged exposure to low temperatures (Trevors et al., 2012). Cold shock proteins are transcriptional anti-terminators or translational enhancers that destabilize RNA secondary structure at low temperatures (Nakaminami et al., 2006). This destabilization of RNA secondary structure prevents premature transcription termination during cold shock. Cold acclimation proteins have been found to have a high level of amino acid sequence identity to members of the CspA family of *E. coli* and the homologous proteins of other microorganisms (Berger et al., 1997). CapA contained highly conserved ribonucleoprotein (RNP1 and RNP2) involved with binding to single stranded DNA and RNA, suggesting similar functionality to Csp proteins.

The expression level of a cold shock protein from *P. fluorescens* MTCC 103 (MW 14 kd) and a cold resistant protein (MW 35kd) from the *P. fluorescens* mutant CRPF8 were studied over a range of temperatures from 37°C down to 4°C (Khan et al., 2003). Expression of the cold shock protein and the cold resistant protein increased in response to decreased temperature with the rate of induction of protein synthesis reaching its maximum at 10°C. Eighteen *Pseudomonas* strains isolated from Antarctica were analyzed for the presence of cold shock proteins and cold accumulation proteins (Panicker et al., 2010). CapB was present in all Antarctic *Pseudomonas* isolates, but CspA was absent. The survival of these isolates in the perennially cold Antarctic environment could be attributed to the continuous expression of CapB and its regulatory role in transcription and translation of essential genes.

### Antifreeze Proteins

Antifreeze proteins also called ice structuring proteins are ice-binding proteins produced by certain organisms to enhance survival in freezing temperatures. Antifreeze proteins inhibit the spread of ice by lowering the freezing point and preventing ice recrystallization (Venketesh and Dayananda, 2008). Antifreeze activity is theorized to occur by an adsorption inhibition mechanism where antifreeze proteins bind to ice crystals and curved ice structures form between the antifreeze proteins. Due to the Kelvin effect, the curved surface is energetically less favorable than flat surfaces which prevents additional water molecules from joining the ice formation (Wang et al., 2017). Ice recrystallization, where ice crystals gradually grow larger in size at the expense of smaller ice crystals, is a lethal stress for cells in frozen conditions. Antifreeze proteins can prevent recrystallization by inhibiting water molecules from leaving ice crystals or by acting as a surfactant to reduce surface tension.

The freezing resistance of antifreeze proteins was discovered in several cold dwelling *Pseudomonas* species including strains of *P. ficuserectae, P. fluorescens, and P. putida* (Kawahara et al., 2004; Singh et al., 2014; Cid et al., 2016). Freeze sensitive mutants and a freeze resistant wild type strain of the plant growth-promoting rhizobacterium *P. putida* GR12-2 were tested for the ability to secrete antifreeze proteins (Kawahara et al., 2001). The freeze sensitive mutants secreted a much lower concentration of antifreeze protein compared to the wild type. Freeze resistance could be partially restored by adding purified antifreeze proteins to the freeze sensitive mutant’s cell suspension. The antifreeze gene *afpA* from *P. putida* GR12-2 was cloned into *E. coli* yielding a 72 kDa protein that exhibited low levels of antifreeze and ice nucleation activities compared to the native protein (Muryoi et al., 2004). The recombinant protein may not have been properly post translationally modified with previous work indicating the carbohydrate lipoglycoprotein was required for ice nucleation activity. These studies demonstrate the potential of accumulated antifreeze proteins to inhibit ice formation in both freeze resistant and freeze sensitive strains.

### Membrane Composition

Altering membrane composition is a critical strategy to combat the rigidity of the cell membrane during cold shock. Membrane fluidity is maintained by increasing the ratio of unsaturated fatty acids to saturated fatty acids or by altering the levels of *anteiso* branched chain fatty acids in membrane phospholipids (Shivaji and Prakash, 2010). Rigidity of the membrane signals the adjustment to membrane lipid composition through biosynthetic pathways including FabAB and Des mediated pathways. The *cis* configuration of unsaturated fatty acids has double bonds which produce a bend in the fatty acid chain (Heipieper et al., 2003). These bends create more space between the unsaturated fatty acid chains producing a more flexible and fluid membrane during cold stress.

The synthesis of unsaturated fatty acids by fatty acid desaturase enzymes is essential for the survival of *Pseudomonas* during cold stress. The cold tolerant strains *Pseudomonas* sp. AMS8 and *Pseudomonas* sp. A3 were found to produce large amounts of monounsaturated fatty acids at low temperatures (Garba et al., 2016, 2018). Δ9- fatty acid desaturase genes were isolated from each of the strains and were subsequently cloned and successfully expressed in *E. coli*. In both studies the recombinant *E. coli* had a functional putative Δ9-fatty acid desaturase capable of increasing the total amount of cellular unsaturated fatty acids. Functional genomics was used to study the cold stress response in the bacterial model organism *P. putida* KT2440 (Frank et al., 2011). Transcriptome sequencing and proteome peptide profiling of KT2440 revealed that the degradation pathway of valine to branched-chain fatty acids (*bkd* operon) was upregulated during growth at low temperatures. These studies illustrate the importance of homeoviscous adaptation to support membrane fluidity during cold stress.

## General Stress Response

**FIGURE 5:**
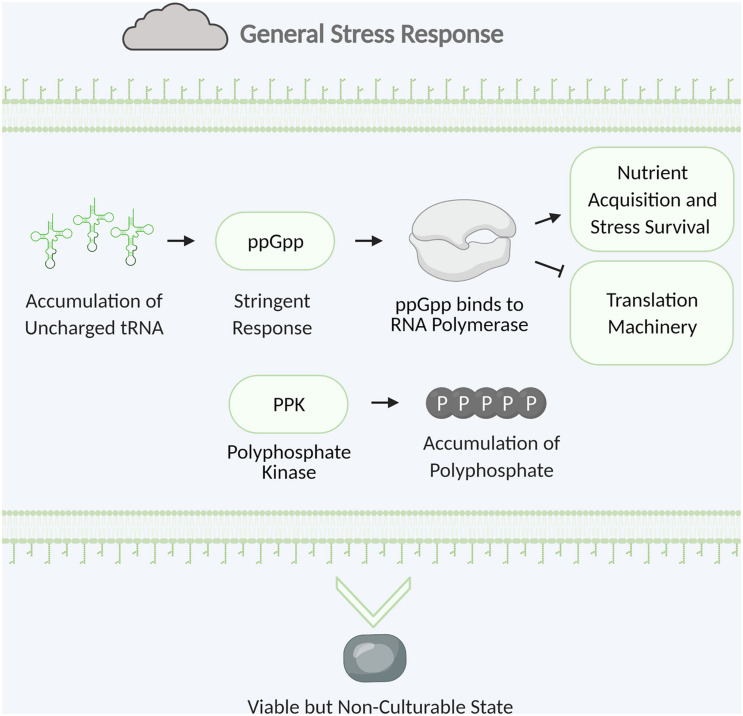
Examples of bacterial general stress response. Stringent response is triggered by the accumulation of uncharged tRNA. The alarmone ppGpp is synthesized and binds to RNA polymerase leading to upregulation of nutrient acquisition and stress survival and downregulation of translation machinery. Polyphosphate kinase catalyzes the formation of polyphosphate which accumulates and is stored for energy. The cell will convert to the viable but non-culturable state with low metabolic activity to survive until hospitable conditions return. Figure created with BioRender.com.

The general stress response is vital to survival in nature when bacteria are challenged with a multitude of different stresses. In the environment bacteria must overcome many stresses including nutrient limitation, desiccation, temperature extremes, and UV irradiation. While targeted stress responses typically help bacteria overcome a specific stress, the general stress response provides resistance against a diverse set of stresses (Storz and Hengge, 2011). General stress response provides cross-protection where defense against one stress can lead to enhanced tolerance to other stresses. This primes the cell for survival against other imminent stresses. The general stress response of *Pseudomonas* species includes transformation into the viable but non-culturable state, storage of polyphosphate, and induction of the stringent response (Figure 5; Storz and Hengge, 2011).

### Viable but Non-culturable State

Viable but non-culturable (VBNC) cells have low metabolic activity and intact membranes, but they are unable to replicate until they enter a more hospitable environment (Ayrapetyan et al., 2018). *Pseudomonas* strains can enter this state as a response to unfavorable environmental conditions such as nutrient limitation, temperature extremes, and desiccation (Lowder et al., 2000; Arana et al., 2010; Pazos-Rojas et al., 2019). Resuscitation methods that allow VBNC cells to repair and transition into a culturable state can vary greatly depending on the type of microorganism and environmental conditions (Bédard et al., 2014). The plant growth-promoting rhizobacterium *P. putida* KT2440 was found to return to a cultivable state after interaction with maize root exudates or a 48 h rehydration in water (Pazos-Rojas et al., 2019). The persistence of VBNC *Pseudomonas* in the natural environment was demonstrated with the biocontrol strain *P. protegens* CHA0 which survived in an uncovered field plot as a combination of dormant and VBNC cells for 72 days (Troxler et al., 2012).

Studies were conducted by Pazos-Rojas et al. (2019) to determine the survival of *P. putida* KT2440 exposed to desiccation stress. *P. putida* KT2440 was subjected to 18 days of desiccation then rehydrated with either maize root exudates for a short time or water for 48 h (Pazos-Rojas et al., 2019). Live/dead staining showed that bacterial counts for desiccated and rehydrated cells were just as high as the counts for the bacteria that did not get exposed to stress. Cells exposed to desiccation stress that were neither rehydrated nor protected with trehalose stained red indicating death or a compromised membrane. The presence of VBNC cells after desiccation exposure was tested again with a GFP-tagged *P. putida* strain which exhibited active GFP expression and housekeeping gene expression was confirmed with RT-PCR. A cytokine secreted by *Micrococcus luteus* called resuscitation-promoting factor (Rpf) has been discovered which enables the resuscitation of VBNC cells in a wide range of Gram negative and Gram positive bacteria (Su et al., 2013). There is limited information on potential homologous genes in *Pseudomonas*, but tests could be conducted to determine if adding Rpf to VBNC *Pseudomonas* cells improves resuscitation.

### Biocontrol Viability Requirements

Biological control agents have a viability standard which is listed on the product label. The company manufacturing the product determines the standard based on product efficacy and typically guarantees a minimum colony-forming unit (CFU) per gram (Cabrefiga et al., 2014; Anderson et al., 2018). The stress of manufacturing live microbial products can lead a fraction of the population to enter a VBNC state (Davis, 2014). VBNC cells can return to a culturable state in more hospitable environmental conditions or by resuscitating microbes through a rehydration process. A large population of VBNC cells which are more difficult to enumerate using traditional plating methods can complicate the process of ensuring the product meets the viability requirements on the label. Two methods to identify culturable and VBNC cells are live/dead staining with microscopic enumeration or detecting housekeeping gene expression by reverse transcription polymerase chain reaction (Li et al., 2014). Understanding the requirements for different metabolic states and improving enumeration techniques are both essential for enabling more accurate viability standards.

### Polyphosphate

Polyphosphate is a polymer containing a few to several hundred phosphate residues linked by high energy phosphoanhydride bonds (Brown et al., 2004). Many bacteria can intracellularly synthesize and degrade polyphosphate with polyphosphate kinases and exopolyphosphatase. Microorganisms accumulate polyphosphate to store energy, and this energy is released by ATP hydrolysis. Polyphosphate is also involved in stress responses including regulation of the stringent response, induction of the stress response sigma factor, and biofilm formation (Nikel et al., 2013; Gray and Jakob, 2014).

The role of polyphosphate was examined with *Pseudomonas* sp. B4 and *P. fluorescens* Pf0-1 polyphosphate kinase deficient mutants. Comparative proteomics on the *Pseudomonas* sp. B4 mutant revealed energy metabolism and nucleoside triphosphate formation were negatively impacted leading to an increase in energy generating metabolic pathways including oxidative phosphorylation and the tricarboxylic acid cycle (Varela et al., 2010). The *P. fluorescens* Pf0-1 mutant was 10-fold less competitive compared to the wild type strain in sterile soil low in inorganic phosphate, and it had an increased sensitivity to elevated temperatures in sterile soil (Silby et al., 2009). These studies demonstrate the importance of polyphosphate in energy storage and stress response for *Pseudomonas* species.

### Stringent Response

Stringent response is a bacterial survival strategy using tRNAs as a sensor to overcome different stressors including nutrient starvation, iron limitation, and heat shock (Vinella et al., 2005; Pletzer et al., 2016). Gene expression switches from growth promotion to survival during stationary phase when nutrients are scarce. The lack of available amino acids leads to an accumulation of ribosomal bound uncharged tRNAs. Binding of uncharged tRNA to the ribosome causes ribosome stalling which triggers RelA synthetase to convert Guanosine-5(’-triphosphate (GTP) into the alarmone guanosine tetraphosphate (ppGpp) (Traxler et al., 2008). SpoT also regulates the stringent response by modifying ppGpp concentration through hydrolysis of ppGpp to guanosine diphosphate (GDP) and pyrophosphate (Boes et al., 2008). ppGpp binds RNA polymerase which halts so that the synthesis of translation machinery (such as ribosomal proteins, rRNA, and tRNA) is decreased, whereas transcription of genes involved in nutrient acquisition, amino acid biosynthesis and stress survival is increased (Wu et al., 2020).

Stringent response is a major factor in stress survival and virulence in both medically and biotechnologically relevant *Pseudomonas* strains. *P. aeruginosa* and *P. putida* stringent response mutants (Δ*relA* Δ*spoT*) were created to test the influence of the stringent response on antioxidant defenses, antibiotic tolerance, biofilm formation, and quorum-sensing (Khakimova et al., 2013; Schafhauser et al., 2014). Studies involving a *P. aeruginosa* mutant revealed that stringent response regulates catalase activity and hydrogen peroxide tolerance during both planktonic and biofilm growth (Khakimova et al., 2013). Another study on a *P. aeruginosa* stringent response mutant showed that (p)ppGpp significantly modulates the quorum-sensing hierarchy of a family of molecules controlling antibacterial and virulence functions (Schafhauser et al., 2014). A *P. putida* stringent response mutant was defective in biofilm dispersal indicating that the stringent response plays a role in relaying the nutrient stress signal to the biofilm dispersal machinery (Díaz-Salazar et al., 2017).

Stringent response has been found to influence the production of secondary metabolites in agriculturally relevant *Pseudomonas* strains. *Pseudomonas* species produce antifungal compounds such as pyrrolnitrin and phenazine that inhibit fungal plant pathogens by suppressing mycelial growth (Huang et al., 2018). Stringent response mutants of the biological control agents *Pseudomonas* sp. strain DF41 and *Pseudomonas chlororaphis* PA23 were created to stop the production of (p)ppGpp (Manuel et al., 2011; Manuel et al., 2012). The *P. chlororaphis* PA23 *relA* mutant had elevated pyrrolnitrin production while phenazine (PHZ) levels remained unchanged. Both *P. chlororaphis* PA23 and *Pseudomonas* sp. strain DF41 *relA* mutants exhibited increased antifungal activity against the pathogen *Sclerotinia sclerotiorum* (Lib.) de Bary. Expression of secondary metabolites involved in antifungal activity are energetically costly and are reduced during the stringent response when nutrients are scarce (Manuel et al., 2012).

### Stress Induced Tolerance

Exposure to sublethal stress is a strategy that has been used to induce the stress response and improve survival during formulation. *Pseudomonas* biocontrol agents have undergone hyperosmotic adaptation to improve formulation viability and efficacy against pathogens (Cabrefiga et al., 2014; Wang et al., 2020b). Comparative transcriptomic analysis was conducted comparing *P. protegens* SN15-2 grown under hyperosmotic conditions or normal osmotic conditions then shocked with lethal temperatures (Wang et al., 2020a). The strains were exposed to cold or heat shock from a range of –15°C to 58°C. Exposure to the hyperosmotic environment increased the cytoplasmic concentration of potassium ions, altered the composition of the cell envelope, and increased trehalose and proline synthesis. The accumulation of potassium ions could be a strategy to maintain the osmotic or turgor pressure after osmotic shock (Cagliero and Jin, 2013). Osmotic stress damaged the cell envelope due to salt-induced dehydration leading to an increase in glycerophospholipid metabolism to maintain membrane integrity (Wang et al., 2020a, b). Triggering the stress response with osmotic stress prepared P. protegens SN15-2 for exposure to other stresses such as lethal temperatures.

This strategy of preparing cells with a sublethal shock translates to industrial formulation stress. Stress adaptation by exposure to osmotic shock has been conducted on *P. fluorescens* EPS62e and *P. protegens* SN15-2 with improved viability after spray drying and fluidized-bed drying, respectively (Cabrefiga et al., 2014; Wang et al., 2020b). Intentionally triggering the stress response or targeting expression of specific survival mechanisms could enhance survival and colonization in the environment leading to better product performance. As described earlier in this review, Manuel et al. (2012) found that induction of stringent response can decrease the expression of some secondary metabolites involved in antifungal activity. When attempting this method, there needs to be an understanding of what mechanisms of survival are induced and if this will lead to a decrease in the production of the antifungal compound of interest.

## Conclusion and Future Directions

*Pseudomonas* species are subjected to stress in the natural environment and have adapted mechanisms to survive these harsh conditions. Members of the *Pseudomonas* genus have been studied for their importance in bioremediation, plant pathogen control, antibiotic resistance, and pathogenicity against humans (Chin-A-Woeng et al., 2003; Folkesson et al., 2012; Wasi et al., 2013). Advances in next generation sequencing and previous work establishing the stress mechanisms of model organisms has accelerated the analysis of stress mechanisms in these important organisms (Ramos et al., 2001; Vorob’eva, 2004). Desiccation, heat, and cold stress can critically impact cells through protein and nucleic acid damage, disruption of major biosynthesis pathways, and loss of membrane integrity.

Bacteria have evolved a number of processes aimed at surviving or thriving under stressful conditions. There are both universal and targeted cellular responses designed to help cells cope with a variety of different stressors. Compatible solutes and polyphosphate can be accumulated as an energy source in the cell for use under critical conditions. Biofilm and EPS production protects the cell through an increased number of persister cells and an extracellular matrix barrier. The viable but non-culturable state is a strategy to persist through harsh conditions by keeping living cells at a low rate of metabolic activity. Chaperones, proteases, thermosensors, and alternative sigma factors rapidly respond to stress by controlling protein damage and regulating stress responses. In response to low temperatures, cold shock proteins and antifreeze proteins prevent premature transcription termination and lower the freezing point, respectively. Bacterial membrane composition changes in response to temperature stress to maintain a flexible and fluid membrane. Knowledge of bacterial stress survival has led to the incorporation of bacterial protectants in the formulation of beneficial microbial products.

New techniques are being established as the knowledge of stress survival mechanisms and availability of genome sequencing are becoming more accessible. Methods demonstrated on model organisms can be applied to industrially relevant *Pseudomonas* strains. Two techniques that have been demonstrated on model microorganisms are selective adaptation and genetic engineering (Billi et al., 2000; Sleight and Lenski, 2007). Selective pressure has been used in laboratory settings to adapt microorganisms to stress and select for more durable phenotypes. *E. coli* populations were subjected to over a hundred cycles of freezing and thawing. After many generations of adaptation to this stress the fitness of the *E. coli* strain was improved by 90% (Sleight and Lenski, 2007). Adaptation is a tool that can be explored in more industrial relevant microorganisms although adaptation can also lead to undesirable traits. Microorganisms can be directly modified for improved resilience to abiotic stresses through genetic engineering. A sucrose-6-phosphate synthase gene from a resilient cyanobacterium was transformed into *E. coli* cells (Billi et al., 2000). Freeze drying, air drying, and desiccation survival was improved 10,000-fold in the transformed cells compared to the wild type cells. Studying the mechanisms of stress survival and trying new techniques to enhance survival is critical for improving beneficial applications and controlling pathogens.

## Author Contributions

All the authors contributed to the concept and ideas of the review, read and approved the submitted version. KC wrote the manuscript and created the figures. BJ and AG critically reviewed and revised the manuscript for publication.

## Conflict of Interest

KC and BJ are employed by the company AgBiome Inc. The remaining author declares that the research was conducted in the absence of any commercial or financial relationships that could be construed as a potential conflict of interest.
